# Galectin-3 Stimulates Tyro3 Receptor Tyrosine Kinase and Erk Signalling, Cell Survival and Migration in Human Cancer Cells

**DOI:** 10.3390/biom10071035

**Published:** 2020-07-11

**Authors:** Nour Al Kafri, Sassan Hafizi

**Affiliations:** School of Pharmacy and Biomedical Sciences, University of Portsmouth, Portsmouth PO1 2DT, UK; nour.al-kafri@port.ac.uk

**Keywords:** Galectin-3, Tyro3, Axl, MerTK, Gas6, protein S, receptor tyrosine kinase, cancer, ligand, signal transduction

## Abstract

The TAM (Tyro3, Axl, MerTK) subfamily of receptor tyrosine kinases (RTKs) and their ligands, Gas6 and protein S (ProS1), are implicated in tumorigenesis and chemoresistance in various cancers. The β-galactoside binding protein galectin-3 (Gal-3), which is also implicated in oncogenesis, has previously been shown to be a ligand for MerTK. However, the selectivity of Gal-3 for the other TAM receptors, and its TAM-mediated signalling and functional properties in cancer cells, remain to be explored. The present study was aimed at determining these, including through direct comparison of Gal-3 with the two canonical TAM ligands. Exogenous Gal-3 rapidly stimulated Tyro3 receptor phosphorylation to the same extent as the Tyro3 ligand ProS1, but not Axl, in the cultured human cancer cell lines SCC-25 (express both Tyro3 and Axl) and MGH-U3 (express Tyro3 only). Gal-3 also activated intracellular Erk and Akt kinases in both cell lines and furthermore protected cells from acute apoptosis induced by staurosporine but not from serum-starvation induced apoptosis. In addition, Gal-3 significantly stimulated cancer cell migration rate in the presence of the Axl blocker BGB324. Therefore, these results have shown Gal-3 to be a novel agonist for Tyro3 RTK, activating a Tyro3-Erk signalling axis, as well as Akt signalling, in cancer cells that promotes cell survival, cell cycle progression and cell migration. These data therefore reveal a novel mechanism of Tyro3 RTK activation through the action of Gal-3 that contrasts with those of the known TAM ligands Gas6 and ProS1.

## 1. Introduction

Tyro3, Axl and MerTK (TAM) are members of a subfamily of receptor tyrosine kinases (RTKs), characterised by homology in terms of gene origins and genomic organisation, amino acid sequence identity and homologous protein domain compositions [1,2]. Furthermore, the TAMs share in common two homologous ligands, namely the vitamin K-dependent proteins Gas6 and protein S (ProS1) [3]. Many studies to date have elucidated the ligand-mediated signalling pathways and functional effects upon activation of the TAM receptors. This includes cancer studies, where each of the TAMs has been shown to be involved in the pathobiology of different tumour types, through aberrant activation in either ligand-dependent or -independent manners [4,5]. The canonical TAM ligands have different affinities for each TAM receptor, Gas6 being able to activate all three TAMs whereas ProS1 activates only Tyro3 and MerTK [3,4]. Nevertheless, both ligands are thought to interact with the receptors through similar contact sites within their homologous domains. 

Much remains to be discovered about Tyro3 RTK in terms of its ligand sensitivity, activation mechanisms and downstream signalling pathways. Recently, we reported that ProS1 is the preferred ligand for Tyro3 receptor in human cancer cells that express multiple TAMs, and identified two discrete signalling axes, ProS1-Tyro3-Erk and Gas6-Axl-Akt, that exist side by side in these cells, mediating proliferative and survival signalling, respectively [6]. However, in addition to the two canonical TAM ligands, evidence has recently been emerging for new non-canonical ligands for the TAM receptors, interacting through different protein–protein interfaces. One of these novel ligands is galectin-3 (Gal-3), for which MerTK was identified as one of several glycan-conjugated novel binding partners [7]. Gal-3 activation of MerTK was shown to stimulate the phagocytic activity of macrophages [7,8]. However, the biophysical nature of the Gal-3–MerTK interaction was not determined, nor is it known whether Gal-3 could also be a ligand for the other TAM receptors. 

As with the other members of the evolutionarily conserved galectin class of proteins, Gal-3 possesses a specific carbohydrate-recognition domain (CRD) through which the protein binds to glycoconjugates containing β-galactosides [9]. However, a unique feature of Gal-3 is its chimeric composition of three structural domains: an N-terminal domain composed of a short leader sequence, a collagen-like internal repeating domain rich in glycine, tyrosine and proline, and the C-terminal CRD. Gal-3 is expressed by many types of human cells including epithelial and immune cells, and also shows a wide sub-cellular distribution [10]. This wide distribution of Gal-3 and its interactions with multiple protein-linked glycans underlie its reported involvement in various cellular processes such as cell differentiation, cell growth, cell apoptosis, cell adhesion, cell cycle regulation, cell migration, immune activation, angiogenesis and chemoattraction [11].

Aside from its functions in the normal state, a pathological role for Gal-3 in contributing to cancer progression has been increasingly uncovered. Elevated levels of Gal-3 observed in leukaemia, lymphomas, breast and thyroid cancer are prognostic for poor patient survival [10]. Additionally, overexpression of Gal-3 has been also reported in multiple cancers including melanoma, head and neck cancer, colorectal, lung, breast and prostate, and Gal-3 was shown to promote tumour growth and protect cancer cells from apoptosis [12]. Amongst its cancer-related molecular interactions, Gal-3 has been shown to be a ligand for the RTKs epidermal growth factor receptor (EGFR) and vascular endothelial growth factor receptor-2 (VEGFR-2), activating signalling pathways for cell proliferation and angiogenesis respectively [13].

In the present study, we have demonstrated that Gal-3 is a novel functional agonist for Tyro3 RTK in human cancer cells and have compared it directly against both well-established TAM ligands Gas6 and ProS1. We have shown that Gal-3 selectively activates Tyro3 but not Axl, and in a distinct manner to the other ligands, uncovering versatility to Tyro3 agonist responsiveness and the intracellular signalling pathways it couples to. Furthermore, we show that Gal-3 promoted cell survival, and stimulated anti-apoptotic gene expression as well as cancer cell migration.

## 2. Materials and Methods

### 2.1. Cell Culture

The human head and neck squamous cell carcinoma cell line SCC-25 (head and neck squamous cell carcinoma; ATCC), expressing both Tyro3 and Axl RTKs, and the human bladder cancer cell line MGH-U3 (gift from Prof. Margaret Knowles, University of Leeds), expressing Tyro3 as sole TAM receptor, were maintained in complete culture medium (DMEM + 10% FBS) at 37 °C in a humidified incubator with 5% CO_2_ as previously described [6].

### 2.2. Cell Treatments

Cells were first serum starved for 24 h, then treated with recombinant human Gas6 developed in-house [14], recombinant human ProS1 (7.5 nM) (Cambridge Protein Works, Cambridge, UK) [6] or recombinant human galectin-3 (Gal-3; 0.2 μM) (Cell Guidance Systems, Cambridge, UK). In some experiments, staurosporine (Sigma Aldrich, Gillingham, UK; 0.1 μM) was added to cells to trigger apoptosis over a 20 h incubation period, in the presence or absence of test ligands.

### 2.3. RNA Extraction and Quantitative Real-Time Polymerase Chain Reaction

Total RNA was isolated from untreated and treated (24-h incubation) cells using the RNeasy Mini Kit (Qiagen, Hilden, Germany) according to the manufacturer’s protocol. In the last step, RNA was eluted with RNAse-free sterile water (40 μL). cDNA was synthesised using the High-Capacity cDNA Reverse Transcription Kit (Applied Biosystems, CA, USA) with 1 μg of RNA template in a 20μl reaction. Real-time PCR was performed for each gene using pre-designed assays containing specific primers and fluorescent hydrolysis probes (Integrated DNA Technologies, Belgium) (Table S1). The experiments were performed using the FastStart Essential DNA Probes Master reaction mix (Roche, Burgess Hill, UK) and run on a LightCycler^®^ 96 System machine (Roche). The amplification procedure entailed 45 cycles of 95°C for 10 s followed by 60 °C for 30 s. For each reaction, *GAPDH* was utilised as the endogenous control gene. The average mRNA fold change in drug-treated samples was normalised against untreated samples using the 2^-∆∆CT^ method [14]. Three independent experiments were carried out and all samples were run in triplicates in each experiment.

### 2.4. SDS-PAGE and Western Blotting

Cells were lysed in ice-cold RIPA buffer (150 mM NaCl, 1% Triton X-100, 0.5% sodium deoxycholate, 0.1% SDS, 50 mM Tris pH 8.0) supplemented with a cocktail of protease and phosphatase inhibitors. Cell lysates were subjected to sodium dodecyl sulphate polyacrylamide gel electrophoresis (SDS-PAGE). The separated proteins were transferred by a wet transfer method onto an activated polyvinylidene fluoride membrane (Millipore, Nottingham, UK). Membranes were incubated for 1 h at room temperature in blocking buffer, which was either Tris-buffered saline-Tween 0.1% (TBS-T; Fisher Scientific, Loughborough, UK) containing 3% non-fat dry milk, or otherwise containing 3% bovine serum albumin (BSA; Fisher Scientific) if probing for phosphorylated proteins. Western blotting was performed on extracts using specific antibodies to detect activated, phosphorylated, forms of Tyro3, Axl, Erk and Akt, as well as GAPDH as a protein loading control, as previously described [6]. The primary antibodies (and dilutions) used were: phospho-Tyro3 (rabbit polyclonal; 1:1000; Sigma) phospho-Axl (rabbit polyclonal; 1:500; R&D systems, Minneapolis, MN, USA), phospho-Erk (mouse monoclonal 1:1000; Cell Signaling Technology (CST), London, UK), phospho-Akt 1/2/3, phospho-Tyro3 (rabbit polyclonal; 1:1000; Sigma), β-actin (rabbit polyclonal; 1:5000; CST), Gal-3 (goat polyclonal; 1:1000; R&D systems) and GAPDH (mouse monoclonal 1:1000; Santa Cruz, Dallas, TX, USA). Secondary antibodies used were donkey anti-rabbit HRP (1:2000; Dako, Denmark), anti-goat HRP (1:5000; Dako) and anti-mouse HRP (1:5000; Promega, Southampton, UK). To produce blots of the best quality, blots were probed for total protein loading through probing for GAPDH, as we have done previously [6], after having first ensured that total Tyro3/Erk/Akt protein levels do not change over the stimulation period that we used in our experiments (Figures S1 and S6). The software *ImageJ* was used for densitometric quantification of Western blot band intensities [15].

### 2.5. Cell Survival/Growth Assay

The effects of various treatments on cell survival/growth were determined by measuring the reduction in [3-(4,5-dimethylthiazol-2-yl)-5-(3-carboxymethoxyphenyl)-2-(4-sulfophenyl)-2H tetrazolium] (MTS) compound (CellTiter 96 Aqueous, Promega) in the presence of phenazine methosulphate (PMS) (Sigma). Cells were seeded in 96-well plates and incubated overnight, prior to indicated treatments for various periods, after which MTS (0.4 μM) was added to cells together with PMS (0.3 nM) and incubated further for 2 h, and absorbance was measured at 490 nm using a spectrophotometric microplate reader (Synergy; BioTek, Potton, UK).

### 2.6. Flow Cytometry

Cells in plates were treated with exogenous proteins Gas6, ProS1 and Gal-3 for 2 h before staurosporine (0.1 μM) was added to trigger apoptosis for a further 20 h. Following treatments, the cells were washed with PBS, trypsinised, collected by centrifugation and re-suspended in 500 μL of binding buffer. The cells were double stained by adding 5 μL of Annexin V-FITC and 5 μL of propidium iodide and incubated at room temperature for 10 min in the dark. Cells were then analysed by flow cytometry using BD FACSCalibur™ (BD Biosciences, New Jersey, NJ, USA) according to a standard procedure (PI: 493 nm (excitation)/636 nm (emission), Annexin V-FITC: 488 nm (excitation)/530 nm (emission)), and the generated data were analysed using *FlowJo-V10* software (BD Life Sciences, Franklin Lakes, NJ, USA).

### 2.7. Scratch Wound Assay

Linear cell migration along a surface was measured by scratch wound assay. A linear scratch was made in a confluent cell monolayer with the end of a 200 μL pipette tip. Images of marked wells were captured at time 0 (when the scratch was made), then again after 21 h, using an inverted live imaging microscope (etaluma 488; Etaluma, San Diego, CA, USA). Image analysis following the experiment was performed using *ImageJ* software [16], and cell migration rates (area change/hour) were calculated.

### 2.8. Statistical Analysis

All data are expressed as the mean ± SEM obtained from a minimum of three independent experiments unless otherwise stated in the figure legend. Quantitative data were analysed by Analysis of Variance (ANOVA) with post-hoc Tukey test for multiple comparisons with one control group or multiple time points/treatments. Statistical analyses and graphical representations were produced using *Prism* software v.8.2.1 (GraphPad Software Inc, San Diego, CA, USA). The level of statistical significance is indicated in the figures and accompanying legends. Differences between groups were considered statistically significant based on the following criteria: * *p* < 0.05, ** *p* < 0.01, *** *p* < 0.001 and **** *p* < 0.0001.

## 3. Results

### 3.1. Galectin-3 is Expressed in a Range of Human Cancer Cell Lines

**Figure 1:**
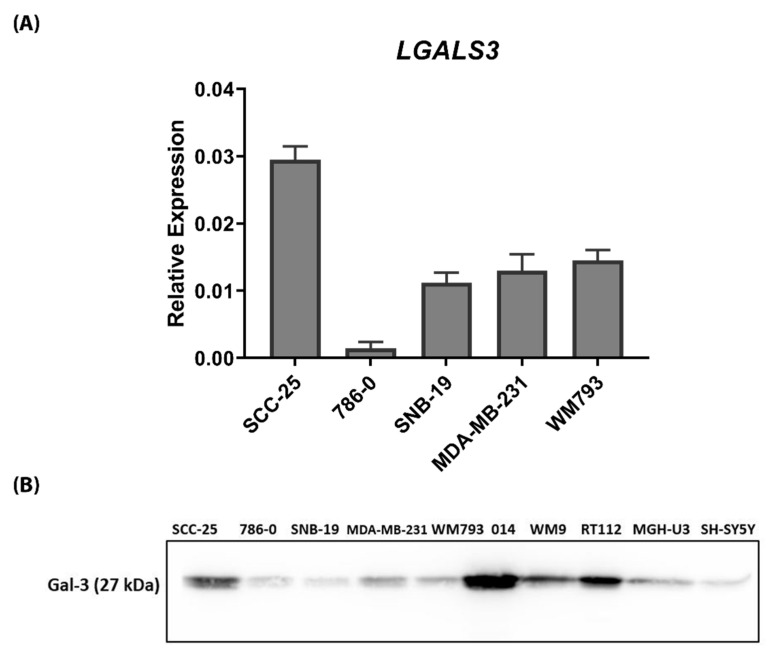
Expression of Gal-3 in human cancer cell lines. (**A**) Quantitative RT-PCR analysis of mRNA expression of Axl, MerTK, Tyro3, Gas6 and ProS1 in five human cancer cell lines. Bars are the mean ± SEM relative gene expression normalised against the housekeeping gene *GAPDH* (*n* = 3 separate experiments). (**B**) Western blot screen of Gal-3 protein expression in ten human cancer cell lines. Equal amounts of total protein were loaded and blot probed with anti-Gal-3 antibody.

We first analysed the expression of Gal-3 in a range of human cancer cell lines at mRNA and protein levels. Gal-3 was widely expressed across the cell line panel, with the expression level ranging from high in melanoma, bladder and head and neck cancer cells, to low in glioma cells (Figure 1).

### 3.2. Galectin-3 Activates Tyro3 But Not Axl

**Figure 2:**
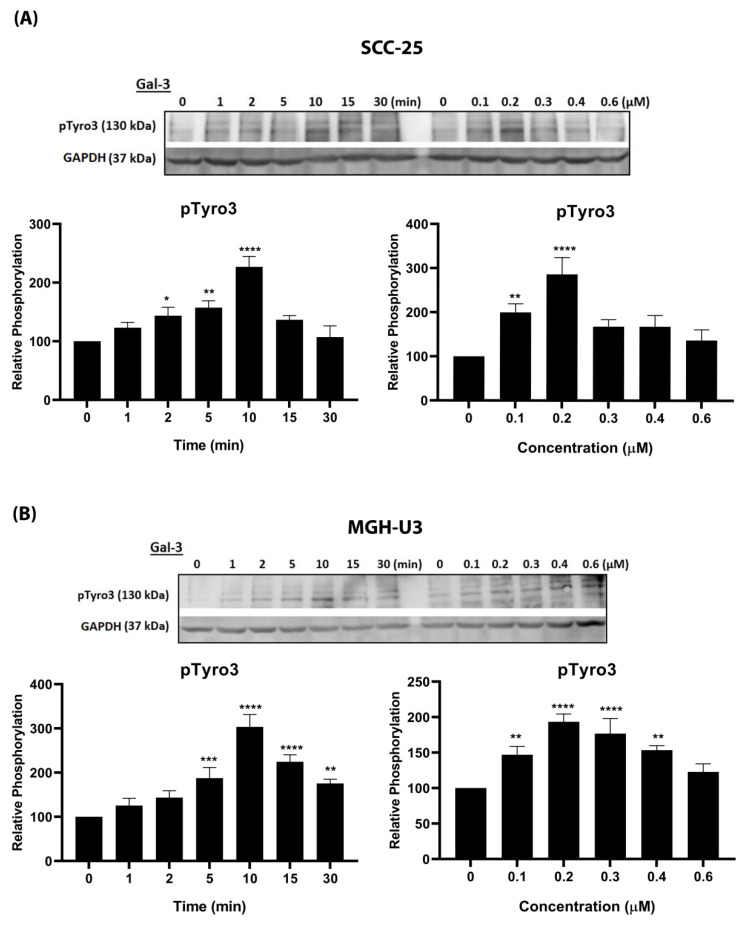
Effect of Gal-3 stimulation on phosphorylation of TAM receptors. (**A**) Representative Western blots showing phosphorylated Tyro3 (pTyro3) protein in SCC-25 (**A**) and MGH-U3 (**B**) cells stimulated by Gal-3 (0.2 μM) in time-course (left) and dose response (right) experiments (9 min stimulation). Accompanying graphs show protein quantification by densitometric analysis of bands. Data are the mean ± SEM protein expression normalised against GAPDH as loading control protein; ANOVA with Tukey’s multiple comparison post-hoc analysis; **** *p*< 0.0001, *** *p* < 0.001, ** *p* < 0.01, * *p* < 0.05 versus control (time 0 or untreated) (*n* = 3 separate experiments).

In order to investigate Gal-3 as a potential TAM ligand, two human cancer cell lines were selected for comparative experiments according to their TAM receptor expression profiles: the head and neck cancer cell line SCC-25, which expresses both Axl and Tyro3, and the bladder cancer cell line MGH-U3, which expresses Tyro3 only. Time-course and dose response experiments with exogenous recombinant human Gal-3 protein were performed, and the phosphorylation levels of both Tyro3 and Axl in SCC-25 cells, and of Tyro3 in MGH-U3 cells, were determined by Western blotting. In both cell lines, Gal-3 rapidly stimulated phosphorylation of Tyro3, peaking at 10 min and decreasing thereafter (Figure 2). Significant Tyro3 activation was observed by Gal-3 from 0.1 μM concentration, with maximal activation occurring at 0.2 μM in both cell lines. As we used GAPDH as a loading control in the blots, we verified that the levels of total kinases did not change over the time periods of the experiments (Figure S1). In contrast to its effect on Tyro3, Gal-3 had no effect on Axl phosphorylation levels in SCC-25 cells over the 15 min time period of study (Figure S2).

**Figure 3:**
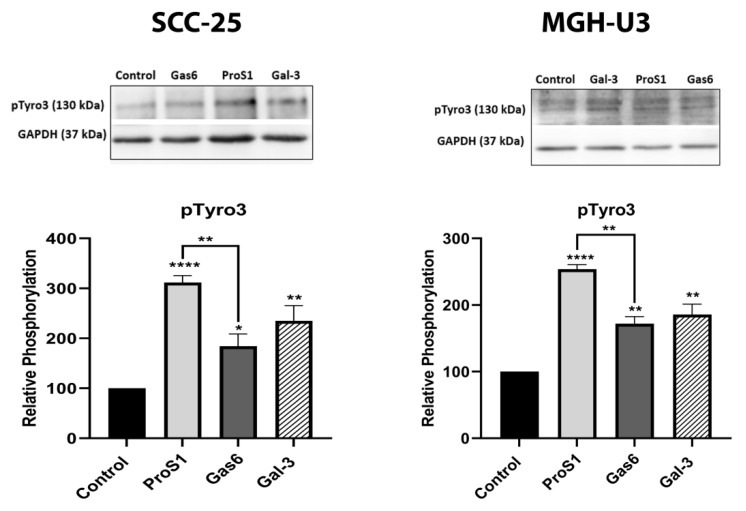
Effects of Gal-3 and the canonical TAM ligands, ProS1 and Gas6, on stimulation of phosphorylation of Tyro3 RTK in human cancer cells. Western blot showing Tyro3 phosphorylation (pTyro3) in serum-starved SCC-25 cells and MGH-U3 cells in response to addition of Gas6 (5.7 nM), ProS1 (7.5 nM) or Gal-3 (0.2 μM) for 9 min. Data are the mean ± SEM protein expression normalised against loading control protein GAPDH (*n* ≥ 3 experiments). ANOVA with Tukey’s multiple comparison post-hoc analysis. * *p* < 0.05, ** *p* < 0.01, **** *p* < 0.0001 and ns, not significant vs. the control or between treatments as indicated by lines.

Next, the TAM receptor-activating effects of Gal-3 were directly compared against those of the two canonical TAM ligands, Gas6 and ProS1, using Gal-3 at 0.2 μM concentration and 9 min stimulation time as optimal experimental conditions from the previous observations. All three exogenous proteins significantly stimulated phosphorylation of Tyro3 in both SCC-25 and MGH-U3 cell lines (Figure 3). ProS1 was the strongest ligand, stimulating Tyro3 roughly twice as much as both Gas6 and Gal-3. Nevertheless, Gal-3 stimulated Tyro3 to at least the same extent as Gas6. In contrast, Axl in SCC-25 cells was stimulated only by Gas6 (Figure S3). Therefore, these experiments show that Gal-3 selectively stimulates Tyro3 but not Axl.

### 3.3. Gal-3 Activates Both Erk and Akt Signalling in Human Cancer Cells

**Figure 4:**
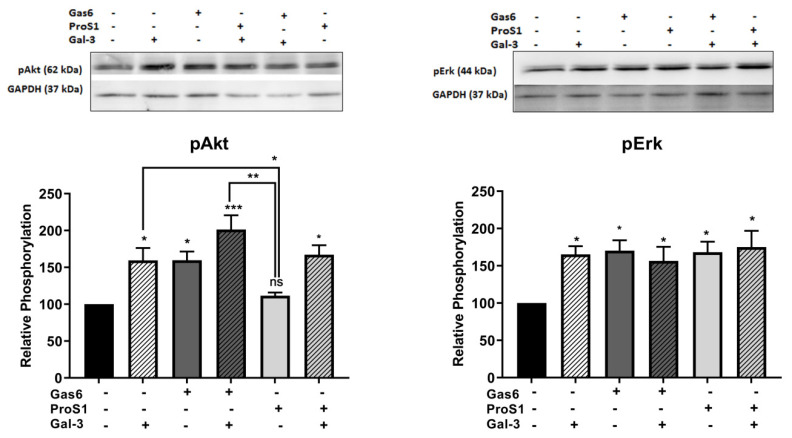
Representative Western blots showing levels of phosphorylated Akt (pAkt) and Erk (pErk) proteins in SCC-25 cells, following addition of Gas6 (5.7 nM), ProS1 (7.5 nM) and Gal-3 (0.2 μM), either alone or in combination, as indicated by the labelling. Densitometric quantification is shown below. Data are the mean ± SEM protein expression normalised against the loading control protein GAPDH (*n* = 3 experiments). ANOVA with Tukey’s multiple comparison post-hoc analysis. * *p* < 0.05, ** *p* < 0.01, *** *p* < 0.001 and ns, not significant vs. the control or between treatments indicated by lines.

As with the receptors, Gal-3 was also compared against the two canonical TAM ligands for their capacity to stimulate intracellular Erk and Akt signalling kinases in SCC-25 cells. Ligands were added in experiments alone or in combination. All three proteins rapidly stimulated Erk1/2 phosphorylation by two-fold (Figure 4). However, Akt phosphorylation was stimulated by Gas6 and Gal-3 but not by ProS1, the latter observation confirming what we have shown before in these cells [6]. 

### 3.4. Gal-3 Protects Cells from Acute Apoptosis by Staurosporine

**Figure 5:**
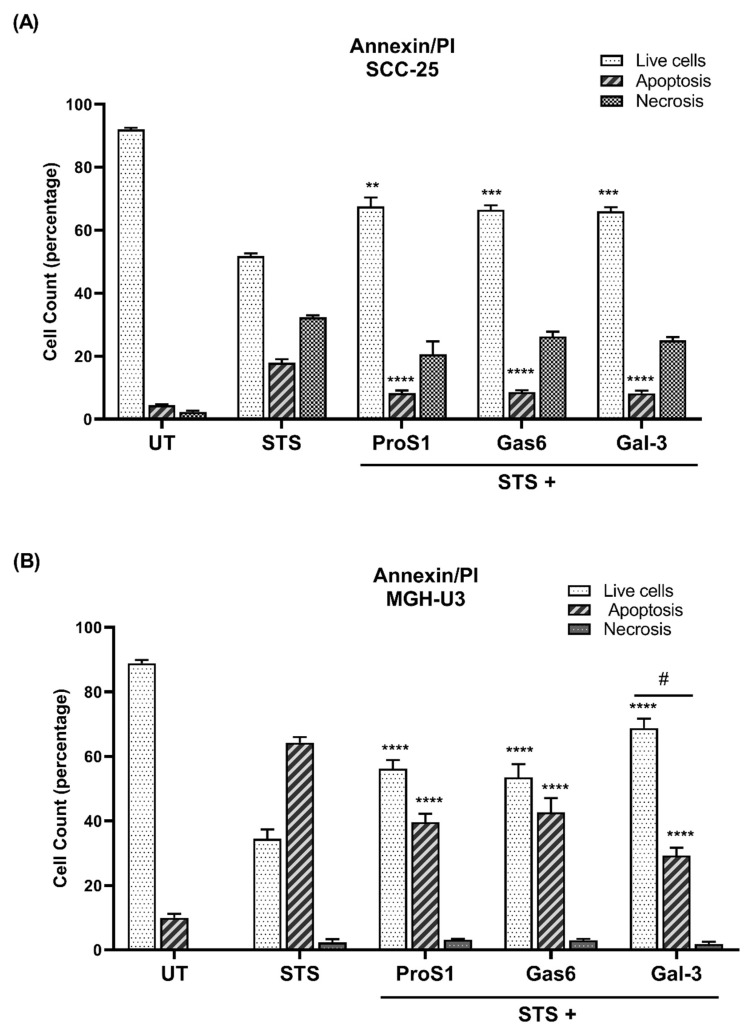
Effect of Gal-3 on cancer cells undergoing apoptosis and role of the TAM receptor expression profile in mediating these effects. Flow cytometry results showing percentage of SCC-25 (**A**) and MGH-U3 (**B**) cells undergoing apoptosis (Annexin V-FITC/PI double-stained) by 20 h incubation with staurosporine (STS; 0.1 μM), with co-incubation with Gal-3 and TAM ligands added 1 h previously. Graphs show proportions (%) of healthy, apoptotic and necrotic cells. Data are the mean ± SEM and underwent ANOVA with Tukey’s multiple comparison post-hoc analysis; **** *p* < 0.0001, *** *p* < 0.001, ** *p* < 0.01 versus the same category of cells in the STS alone treated group (*n* = 3 experiments). (#) indicates the comparisons between Gal-3 cell fraction versus the same category of cells in both Gas6 and ProS1 treatment groups; *p* < 0.05.

Flow cytometry revealed that Gal-3 significantly protected cancer cells from acute apoptosis induced by staurosporine (Figure 5). In both SCC-25 and MGH-U3 cell lines, the presence of Gal-3 together with staurosporine significantly reduced the percentage of cells undergoing apoptosis as compared to treatment with staurosporine alone (Figure 5). Moreover, in MGH-U3 cells, the anti-apoptotic effect of Gal-3 was significantly stronger than that of the two canonical TAM ligands, indicating that Gal-3 could activate receptors/pathways also beyond Tyro3, to which ProS1 and Gas6 were necessarily restricted. In addition, all three proteins significantly protected cells from acute apoptosis by staurosporine also in an MTS assay (Figure S4).

**Figure 6:**
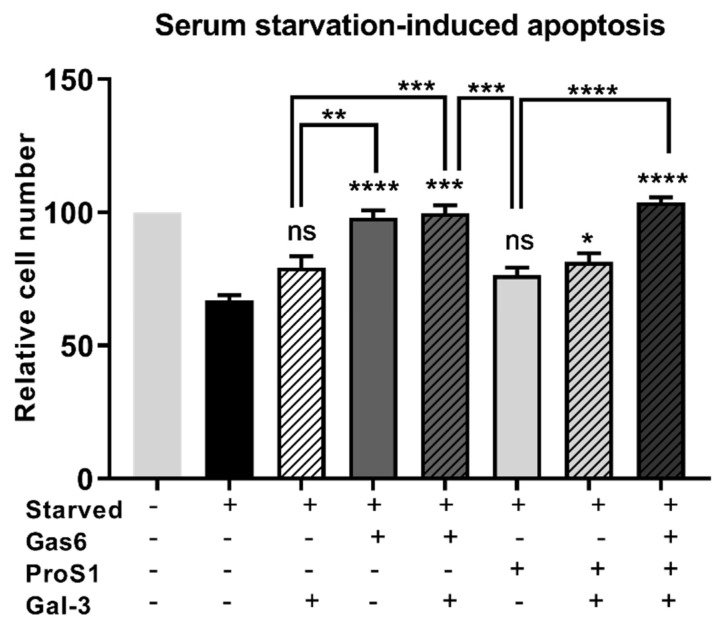
MTS assay of survival of SCC-25 cells treated with Gas6, ProS1 or Gal-3 separately or jointly in a serum-free medium to trigger apoptosis by prolonged serum starvation over 15 days. Data are the mean ± SEM and underwent ANOVA with Tukey’s multiple comparison post-hoc analysis; **** *p* < 0.0001, *** *p* < 0.001, ** *p* < 0.01, * *p* < 0.05 vs. untreated starved cells or for comparisons indicated by lines (*n* = 4 experiments).

As an alternative means by which to induce apoptosis, cells were subjected to long-term serum starvation to determine the effect of Gal-3 on cell viability under those conditions by MTS assay. Under long-term serum starvation conditions, only Gas6 out of the three proteins conferred a significant positive survival effect on cells when used as sole agent (Figure 6). Nevertheless, there was a trend towards significance with Gal-3 when used as sole agent, in terms of counteracting the serum starvation-induced apoptosis. Furthermore, a significant protection from apoptosis was achieved when Gal-3 was combined with ProS1, which also did not have a significant effect alone, indicating an additive effect.

### 3.5. Comparative Effects of Gal-3 vs. TAM Ligands on Genes Regulating Cell Cycle Progression and Apoptosis

**Figure 7:**
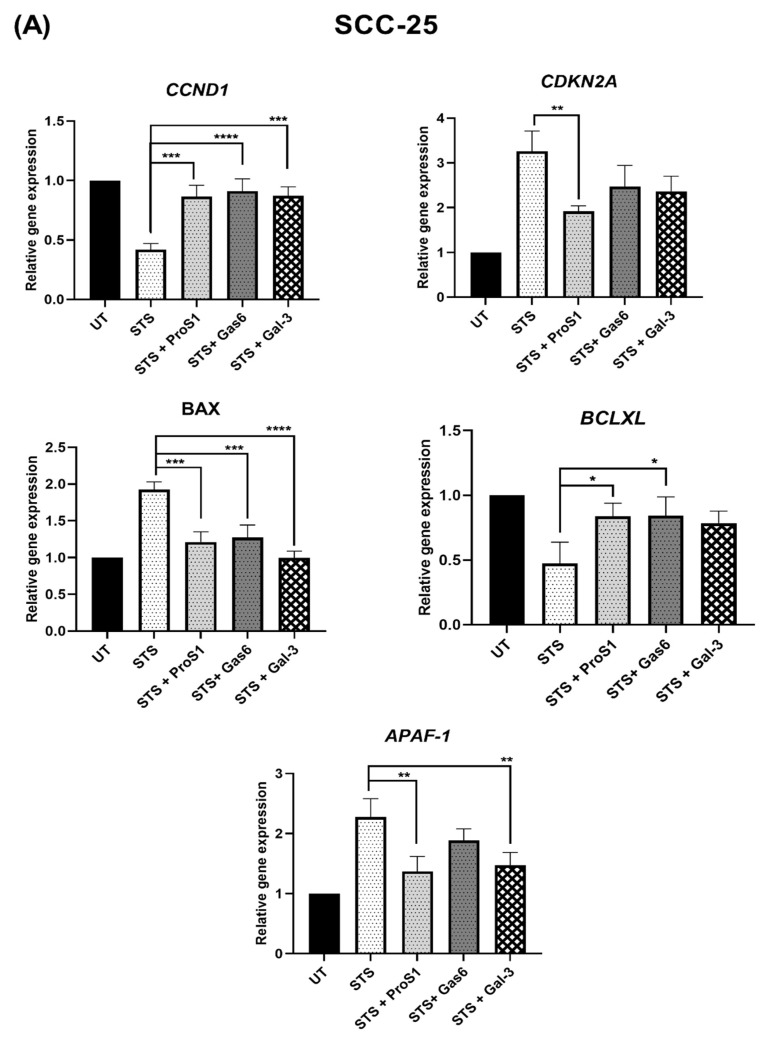

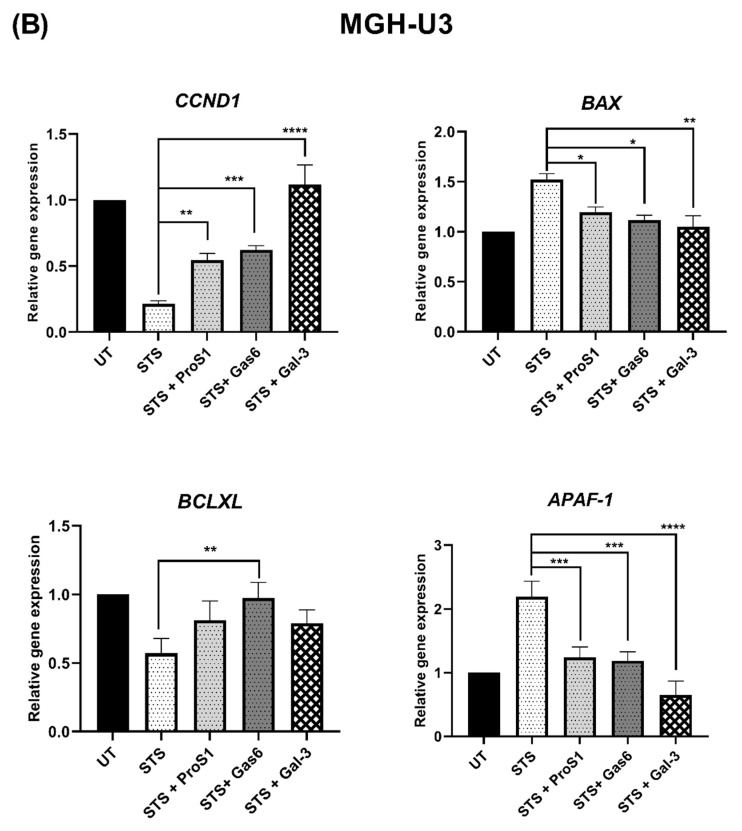
Quantitative RT-PCR analysis of effects of Gal-3 and TAM ligands on the expression of genes regulating apoptosis (*BAX*, *BCLXL*, *APAF-1*) and cell cycle progression (*CCND1*, *CDKN2A*) in SCC-25 (**A**) and MGH-U3 (**B**) cancer cells. Cells were treated for 20 h with staurosporine (STS; 0.1 μM), with co-incubation with Gas6 (5.7 nM), ProS1 (7.5 nM) or Gal-3 (0.2 μM) added 2 h previously. Relative gene expression is represented as fold change, (2^−ΔΔCt^) is the normalised gene expression (2^-ΔCt^) in the treated samples divided by the normalised gene expression (2^−ΔCt^) in the control sample (untreated; UT). Relative gene expression in each sample was normalised against the housekeeping gene *GAPDH*. Data are the mean ± SEM and underwent ANOVA with Tukey’s multiple comparison post-hoc analysis; **** *p* < 0.0001, *** *p* < 0.001, ** *p* < 0.01, * *p* < 0.05 versus STS-treated sample (*n* = 3 experiments).

To build on the observations made on Gal-3 and TAM ligand activation of Erk and Akt signalling in human cancer cells, we analysed the expression of a set of genes well known to regulate apoptosis or cell progression following stimulation with Gal-3 and the two TAM ligands. SCC-25 and MGH-U3 cells were treated with staurosporine for 20 h to induce apoptosis, in the absence or presence of each individual protein, followed by qRT-PCR analysis of each gene. In both cell lines, staurosporine alone caused a strong reprogramming of gene expression in a pro-apoptotic direction, upregulating the pro-apoptotic genes *APAF-1* and *BAX* whilst downregulating the anti-apoptotic gene *BCLXL* (Figure 7). Staurosporine also affected genes leading to cell cycle arrest, including downregulating *CCND1* and upregulating *CDKN2A* gene expression. In both cell lines, co-treatment of staurosporine with each of the three proteins reversed the effect of staurosporine alone, with variations amongst the proteins.

### 3.6. Galectin-3 Stimulates Cancer Cell Migration During Axl Blockade

**Figure 8:**
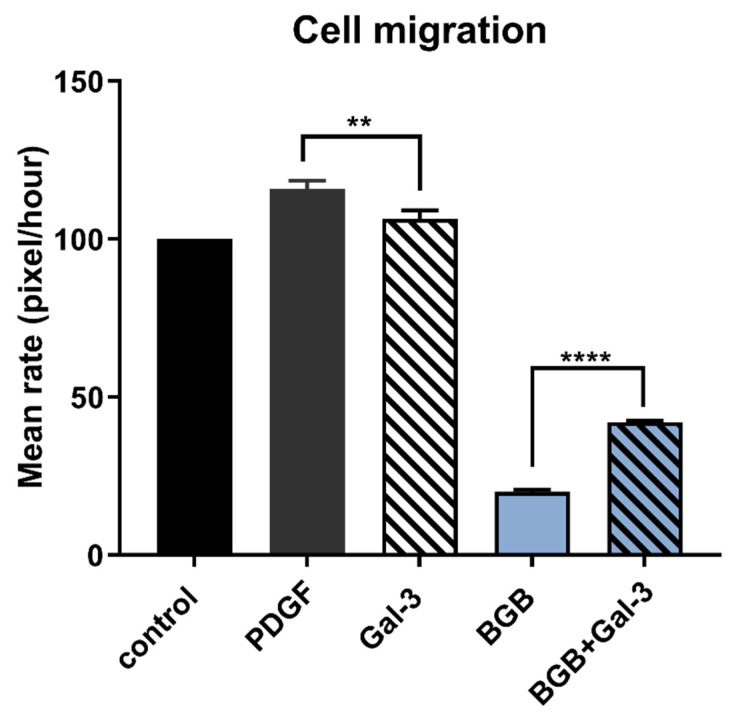
Effect of Gal-3 on cell migration. Cell migration rate of SCC-25 cells in scratch wound assay, expressed as a percentage vs. the control (untreated). Cells were treated with PDGF (10 nM) as a positive control, Gal-3 (0.2 μM), Axl inhibitor BGB324 (BGB; 10 μM) and a combination of both Gal-3 and BGB324. The averages of gap distance at 0 and 21 h and the gap closure rate were calculated for each set of treatments. Data are the mean ± SEM and underwent ANOVA with Tukey’s multiple comparison post-hoc analysis; **** *p* < 0.0001, ** *p* < 0.01 for comparisons indicated by lines (*n* = 4 experiments).

The influence of Gal-3 on the linear migration of SCC-25 cancer cells was measured by “scratch wound” assay combined with time lapse microscopy. Platelet-derived growth factor (PDGF; 10 nM) was used as a positive control for cell migration. Exogenous Gal-3 did not affect cell migration rate alone; however, when Gal-3 was co-incubated with cells together with the Axl inhibitor BGB324, which on its own markedly reduced the rate of cell migration, Gal-3 significantly reversed the reduction in migration due to Axl blockade (Figure 8). This indicates that Gal-3 is able to stimulate cell migration via pathways independent of Axl, which may include Tyro3. 

## 4. Discussion

The homologous vitamin K-dependent proteins Gas6 and ProS1 were first identified as ligands for the TAM RTKs in the mid-1990s [3]. Since then, a long period of time elapsed before evidence was presented for the existence of novel TAM ligands. A study of mediators of phagocytosis identified the proteins Gal-3 and Tubby to bind to and activate MerTK, and TULP-1, which bound all three TAMs [17]. MerTK binding by Gal-3 was suggested to occur through the latter’s C-terminal carbohydrate recognition domain (CRD), which may harbour a MerTK-binding motif found in the two LG domains in ProS1 and Gas6, or the MPD motif of Tubby and Tulp-1 [7]. The presence of such a motif in Gal-3 therefore also laid open the possibility of significant affinity for TAMs other than MerTK. Moreover, given Gal-3′s high affinity for glycans and the likely presence of N-linked glycosylation on the surface of all three TAMs, there was also a possibility of Gal-3 binding to TAMs as a canonical lectin, and thus in a manner distinct from the two vitamin K-dependent TAM ligands.

In the present study, Gal-3 stimulated phosphorylation of Tyro3 in both time- and concentration-dependent manners in both SCC-25 and MGH-U3 cancer cell lines. This was mirrored by Gal-3 stimulation of Erk kinase phosphorylation, and hence there was a similarity with the ProS1-Tyro3 axis which we have previously uncovered [6]. The use of these two cell lines with distinct natural TAM expression profiles (specifically differing in Axl expression) enabled us to demonstrate that Gal-3 has agonistic activity selectively via Tyro3 but not via Axl. A high basal level of Axl activation in these cells could indicate that Gal-3 may have been able to stimulate Axl if Axl expression was lower; however, these cells are still responsive to Gas6 and therefore any agonistic potential of Gal-3 against Axl is likely to be low, alongside glycosylation differences between the TAM receptors, as discussed later. Nevertheless, these data represent the first observation that Gal-3 is an agonist for Tyro3 receptor, and validates the original report by Caberoy *et al*. [7] which identified Gal-3 as a ligand for MerTK. The concentrations of Gal-3 observed to be active here indicate that, as with growth factors, it is likely to be the local concentrations of the protein surrounding the cells within a tumour that determine the efficacy of the protein. 

The TAMs are known to have N-linked glycosylation sites on their surface that could potentially be recognition sites for lectins such as Gal-3. All three TAMs possess multiple asparagine (N) residues within their amino acid sequences (Figure S5) [18]. However, it is clear that Tyro3 and MerTK have more potential glycosylation sites than Axl, as well as there being a higher incidence of similarities in terms of the locations of glycosylation sites in both MerTK and Tyro3 but not in Axl, for example N63 and N293 in Tyro3 and N114 and N354 in MerTK. Therefore, the presence of certain homologous asparagine residues in Tyro3 and MerTK, but their absence in Axl, may explain our observation that Gal-3 stimulated Tyro3 (and MerTK as observed by Caberoy *et al.* [7]) but failed to stimulate Axl. Thus, one or more of these potential N-linked glycosylation sites exclusive to Tyro3 and MerTK could form an interaction interface with Gal-3. 

**Figure 9:**
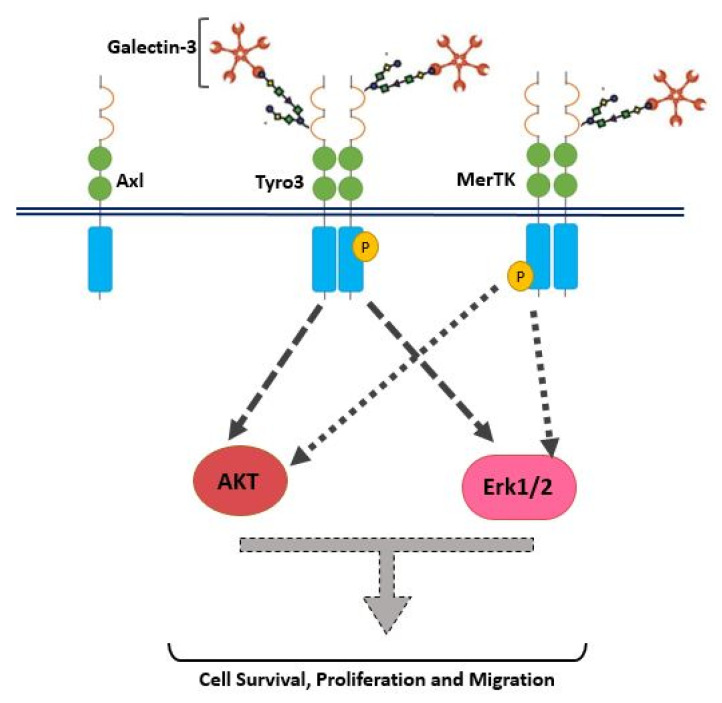
Schematic of Gal-3 as an agonist for Tyro3 in human cancer cells. Gal-3, represented here in its pentameric form as has been reported, stimulates Tyro3 phosphorylation and activation, as well as downstream activation of Erk and Akt signal transduction pathways, which drive cell proliferation and survival/migration, respectively. The illustration of Gal-3 binding directly to a glycosyl chain on Tyro3 is a proposed model of Gal-3 agonistic action and requires investigation.

In addition to receptor selectivity, Gal-3 was observed to activate both intracellular Erk and Akt signalling in SCC-25 cancer cells. This observation distinguishes Gal-3 from ProS1 due to the additional capacity of Gal-3 to activate Akt (which ProS1 lacks), whereas a distinction from Gas6 lies in the observation that Gal-3 failed to stimulate Axl receptor. Therefore, these results may suggest that Gal-3 is connected to the Tyro3-Erk signalling axis as well as, additionally, downstream Akt signalling, although not via Axl but possibly through one or more other RTKs (Figure 9). Our findings here on Tyro3 add to and concur with those on other RTKs, via which Gal-3 has been shown to activate Erk and Akt signalling. This therefore suggests a commonality in the signalling mechanism by which Gal-3 exerts its effects on cancer cells via RTKs. In addition, our findings here reveal similarities and differences between Gal-3 and the two canonical ligands. Gal-3 also protected cells from apoptosis induced by staurosporine (short term) but not after serum starvation (long term), which likens it to ProS1 but not Gas6, which had the most powerful and durable cell survival effect. Therefore, Gal-3 may act via Tyro3 in cells in the same manner as ProS1, which accounts for its more limited cell survival effects as opposed to Gas6, which activates Axl to exert a more pronounced cell survival function [18]. Nevertheless, the Gal-3 effect approached statistical significance in terms of counteracting the serum starvation-induced apoptosis and, moreover, both Gal-3 and ProS1 together were able to stimulate survival-signalling pathways to a sufficient degree through an additive effect. Furthermore, gene expression analysis by RT-qPCR showed that Gal-3 counteracted the apoptosis-inducing and cell cycle arresting effects of staurosporine in cells, therefore emulating the TAM ligands as activators of cell survival and proliferative signalling. 

Further in keeping with its activation of pro-tumorigenic signalling pathways in cancer cells, Gal-3 was also able to stimulate human cancer cell migration. Although Gal-3 was a weak stimulator of migration as sole agent, this observation was likely due to an already high basal rate of migration in these cells that masked the Gal-3 ligand potential. However, Gal-3 had a clearer and pronounced stimulatory effect when the component of cell migration that was mediated through Axl signalling was blocked in the presence of the small molecule inhibitor Axl BGB324. It could alternatively be stated that Gal-3 significantly hindered the suppressive effect of Axl blockade on cell migration. This observation therefore indicates that Gal-3 signals towards cell migration, at least in part, independently of Axl. However, as this signalling is unlikely to occur via Tyro3, the specific receptor-mediated pathway for this remains to be determined. 

Therefore, in this study we have identified Gal-3 as a novel selective agonist/ligand for Tyro3 RTK, which highlights a similarity with the other TAM, MerTK, but a difference with Axl. However, it remains to be observed whether Gal-3 acts as a ligand for Tyro3 through direct physical binding of the receptor; this can be determined through e.g. a receptor binding assay. Furthermore, it is also currently unknown whether Gal-3 interacts with TAM receptors through its lectin properties, i.e. with a glycosyl chain on the RTK, or else via another as yet unidentified interaction interface. Overall, the effects of Gal-3 on e.g. cancer cell survival and migration are likely due to a combination of effects via several cell surface receptors. As Gal-3 is considered as a biomarker for many cancers, often showing increased expression, knowledge on the molecular mechanisms by which it affects cancer cells, e.g. through Tyro3 activation as shown here, it can offer a novel avenue for cancer therapeutics and/or diagnostics. For example, Gal-3′s ability to mediate the clearance of apoptotic cells via MerTK implicates it as a role player in immune resistance and immunosuppression, contributing to conditions such as cancer and autoimmune diseases [19]. Additionally, Gal-3 expression has been used to determine treatment responsiveness, as, for example, negative/low levels of Gal-3 indicate an early and durable response to immunotherapy in lung cancer [20]. Furthermore, other than cancer, Gal-3 shows promise as a biomarker for many other diseases including hepatitis [21], cardiovascular diseases [22], chronic kidney disease and Alzheimer’s disease [23]. In some of these diseases the TAM receptors may also feature, thus potentially indicating involvement of a Gal-3/TAM signalling axis in the underlying mechanisms. Further work is required to elucidate the precise molecular mechanisms surrounding Gal-3-TAM receptor interaction and its downstream functional consequences in different pathologies. 

## Supplementary Materials

The following are available online at https://www.mdpi.com/2218-273X/10/7/1035/s1, Figure S1: Total Tyro3 protein levels do not change over time period of ligand exposure; Figure S2: Effect of Gal-3 on phosphorylation of Axl in human cancer cells; Figure S3: Effects of Gal-3 and the canonical TAM ligands, ProS1 and Gas6, on stimulation of Axl RTK in SCC-25 cells; Figure S4: MTS assay of viability of SCC-25 cells treated with Gas6, ProS1 or Gal-3 separately or jointly in serum-free medium under conditions of apoptosis triggered by 20 h treatment with staurosporine (STS; 0.1 μM); Figure S5: Amino acid sequence alignment of human Tyro3 (NP_006284.2), MerTK (NP_006334.2) and Axl (NP_068713.2) proteins within their extracellular domains using *Clustal Omega* software; Figure S6: Western blots confirming changes in levels of phosphorylated Erk (pErk) and Akt (pAkt) proteins relative to total Erk and Akt, respectively, in the same samples; Table S1: Primer/probe sets used in RT-qPCR.
